# INCIDENCE AND ASSOCIATIONS OF UNPLANNED ACUTE CARE UNIT READMISSIONS OF PRIMARY BRAIN TUMOUR PATIENTS DURING REHABILITATION: A RETROSPECTIVE STUDY

**DOI:** 10.2340/jrm-cc.v8.41974

**Published:** 2025-04-01

**Authors:** Matthew Rong Jie Tay, Justin Desheng Seah, Karen Sui Geok Chua

**Affiliations:** 1 Tan Tock Seng Hospital Rehabilitation Centre, Singapore; 2 Institute of Rehabilitation Excellence (IREx), Singapore; 3 Lee Kong Chian School of Medicine, Nanyang Technological University, Singapore; 4 Yong Loo Lin School of Medicine, National University of Singapore, Singapore; 5 Department of Post Acute and Rehabilitative Care, Woodlands Health Campus, Singapore; 6 Rehabilitation Research Institute of Singapore, Nanyang Technological University, Singapore, Singapore

**Keywords:** brain neoplasms, glioblastoma, length of stay, patient readmission, rehabilitation centres, rehabilitation, neurosurgical procedures

## Abstract

**Objective:**

To examine incidence and associations for unplanned Acute Care Unit Readmissions (ACURs) in Asian primary brain tumour patients.

**Design:**

A retrospective single-centre cohort study.

**Patients:**

A total of 173 Asian primary brain tumour patients undergoing inpatient rehabilitation in a tertiary rehabilitation centre.

**Methods:**

Primary outcome was unplanned ACUR. Logistic regression analysis was used to determine associations with patients who had an unplanned ACUR.

**Results:**

Altogether, the majority of patients had low-grade (World Health Organization Class I and II) tumours (76.9%), whilst 32 (18.5%) patients had glioblastoma multiforme tumours. Unplanned ACUR occurred in 27 (15.9%) patients, with the 2 most common causes being neurosurgical complications (37.0%) and non-neurosurgical infections (25.9%). Significant risk factors for ACUR patients were a longer acute hospitalization stay (odds ratio = 1.024; 95% confidence interval [CI] = 1.01–1.04; *p* = 0.007), whereas a higher admission motor Functional Independence Measure was protective against unplanned ACUR (odds ratio = 0.945; 95% CI = 0.915–0.977; *p* = 0.001).

**Conclusions:**

Despite rehabilitation goals of prevention of complications, patients with primary brain tumours undergoing inpatient rehabilitation continue to demonstrate significant unplanned ACUR rates (15.9%) with neurosurgical complications being common. These findings underscore the importance of continued vigilance, access to and coordination of neurosurgical care and management beyond the acute surgical phase, in order to ensure optimal outcomes.

## LAY ABSTRACT

Inpatient rehabilitation is vital for recovery in patients with primary brain tumours. However, some of these patients may experience medical complications, which require an interruption in their rehabilitation and a transfer to acute hospital. This study aimed to understand how frequent and why Asian patients with primary brain tumours are readmitted to acute care units after starting rehabilitation. In our study, we found 15.9% of patients were unexpectedly readmitted to acute care units. The most common reasons for these readmissions were complications related to brain tumour and infections. Key factors influencing readmission included a longer initial hospital stay, which increased the likelihood of readmission, and a higher motor function score upon admission, which made readmission less likely. Even though rehabilitation aims to prevent complications, primary brain tumour patients still face a notable risk of readmission due to complications. This highlights the need for careful monitoring and ongoing care even during inpatient rehabilitation.

With advancement in neurosurgical and oncological treatment, the survival of patients with primary brain tumours has improved substantially. However, these patients often have long-term impairments, either due to the tumour itself or the effects of treatment, including surgery, radiation and chemotherapy. Due to the disabling nature of primary brain tumours, many patients with brain tumours often require inpatient rehabilitation (1). Although benign tumours are more treatable with a higher survival rate, many of these patients also experience functional deficits due to treatment complications. For example, patients with brain tumours can experience motor deficits, including hemiparesis, gait impairments and incoordination, as well as visual-perceptual and sensory impairments (2). Executive dysfunction and cognitive deficits can also result from residual tumour, disease progression and treatment in these patients, which impact their functioning and quality of life.

Whilst high-quality evidence for the benefits of inpatient rehabilitation in patients with primary brain tumours is currently sparse, it is largely believed that multidisciplinary inpatient rehabilitation is important to address various functional and cognitive deficits in these patients (3). Studies have reported that patients with primary brain tumours undergoing inpatient rehabilitation have functional gains comparable to stroke patients on discharge (4). Unfortunately, patients with cancer have a high rate of unplanned Acute Care Unit Readmissions (ACURs) due to an increased medical complexity and instability, as well as a risk of tumour recurrence or progression (5). However, there are currently limited studies on unplanned ACUR from rehabilitation in Asian settings (6).

Hence, we aimed to study Asian patients with primary brain tumours undergoing inpatient rehabilitation to determine the incidence, causes and associations for unplanned ACUR from inpatient rehabilitation.

## METHODS

### Patients

A retrospective cross-sectional study was performed on all patients with primary brain tumours admitted to the Acquired Brain Injury Rehabilitation unit at the Tan Tock Seng Hospital Rehabilitation Centre during the period from 2013 to 2020.

Patients who were included in our study were admitted for inpatient rehabilitation from acute hospitals. All patients had findings of a primary brain tumour confirmed on computed tomography or magnetic resonance imaging of the brain, in addition to histological confirmation from either brain biopsy or surgical resection. Brain tumours were graded according to World Health Organization (WHO) classification (7). Patients who had a metastatic brain lesion were excluded. Generally, patients with an unplanned ACUR had onset of medical complications that could not be managed in a rehabilitation setting and were transferred back to the referring hospital after phone consultation with the referring clinical team. This study was approved by the institutional review board (NHG DSRB 2020/01088). Waiver of consent was obtained due to the retrospective chart review study design. Findings of functional outcomes from this study cohort had been published previously (3).

### Clinical data and outcome measures

The primary outcome was the incidence for unplanned ACUR, which was obtained from electronic medical records. Causes for unplanned ACUR were grouped into the following categories: Neurosurgical complications, disease progression, infection, seizures and cardiac causes.

The Eastern Cooperative Oncology Group (ECOG) performance status was used to assess the patient’s baseline overall health status. It is a 6-level item administered by clinicians. The score ranges from 0 (fully active, able to carry on all pre-disease performance without restriction) to 4 (completely disabled) or 5 (deceased).

Admission and discharge functional status were assessed during inpatient rehabilitation by a multidisciplinary team using the Functional Independence Measure (FIM) score, a commonly used 18-item measure of functional status grouped into separate motor (13 items) and cognitive (5 items) domains. It evaluates activities of daily living across 6 areas (self-care, sphincter control, transfers, locomotion, communication and social cognition). Each item is scored on a scale ranging from 1 to 7 (dependent to independent). FIM items are then combined into motor and cognitive scores, using the 13 motor items to derive the motor score and the 5 cognitive items to develop the cognitive score.

A motor FIM score range of 13 to 91 and a cognitive FIM score range of 5 to 35 are then obtained. All FIM scores were obtained by trained therapists who were credentialed through the Uniform Data System for Medical Records, within 72 h of inpatient rehabilitation after transfer (i.e. admission) from acute hospitals and discharge.

### Statistical analysis

Differences between groups were tested using the t-test and chi-square test.

Logistic regression analysis was used to determine associations with patients who had an unplanned ACUR, with independent variables being age, sex, ethnicity, marital status, ECOG performance status, lesion side, lesion location, lesion size, grade, tumour recurrence, radiotherapy, chemotherapy, surgical treatment, treatment received during acute hospitalization (steroids and antiepileptic drugs), acute and rehabilitation length of stay, admission FIM motor and cognition scores.

Significance was set at *p* < 0.05. Analysis of the data was performed using SPSS version 26.0 (IBM Corp., Armonk, NY).

## RESULTS

Altogether, there were 173 patients included, with 67 (38.7%) patients aged > 60 years. The mean age was 55.5 (± 13.0) years. The study population was predominantly Chinese (77.5%), with pre-existing ECOG of 0 (41.0%) or 1 (59.0%). Table I shows the baseline characteristics of the sample and differences in the patients with and without unplanned ACUR.

**Table I:** Clinical characteristics

Variable	All patients (*n* = 173)	No. unplanned ACUR (*n* = 27)	Unplanned ACUR (*n* = 146)	*p*
**Age, *n* (%)**				0.017
- < 60	106 (61.3)	11 (40.7)	95 (65.1)	
- > 60	67 (38.7)	16 (59.3)	51 (34.9)	
Age, mean (SD)	55.5 (± 13.0)	58.6 (± 11.5)	54.9 (± 13.2)	0.169
**Sex, *n* (%)**				0.128
- Male	67 (38.7)	14 (51.9)	53 (36.3)	
- Female	106 (61.3)	13 (48.1)	93 (63.7)	
**Race, *n* (%)**				0.294
- Chinese	134 (77.5)	24 (88.9)	110 (75.3)	
- Malay	29 (16.8)	2 (7.4)	27 (18.5)	
- Indian	10 (5.8)	1 (3.7)	9 (6.2)	
**Marital status, *n* (%)**				0.398
- Single	57 (32.9)	7 (25.9)	50 (34.2)	
- Married	116 (67.1)	20 (74.1)	96 (65.8)	
**ECOG performance status, *n* (%)**				0.376
-0	71 (41.0)	9 (33.3)	62 (42.5)	
-1	102 (59.0)	18 (66.7)	84 (57.5)	
**Lesion side, *n* (%)**				0.758
- Left	66 (38.2)	12 (44.4)	54 (37.0)	
- Right	73 (42.2)	10 (37.0)	63 (43.2)	
- Bilateral	34 (19.7)	5 (18.5)	29 (19.9)	
**Lesion site, *n* (%)**				0.121
- Frontal/temporal/parietal	105 (60.7)	21 (77.8)	84 (57.5)	
- Infratentorial	48 (27.7)	5 (18.5)	43 (29.5)	
- Skull base	20 (11.6)	1 (3.7)	19 (13.0)	
**Lesion size (cm), *n* (%)**				0.314
- < 3	38 (22.0)	3 (11.1)	35 (24.0)	
- 3–6	110 (63.6)	19 (70.4)	91 (62.3)	
- > 6	25 (14.5)	5 (18.5)	20 (13.7)	
Grade, *n* (%)				0.004
- Low grade	133 (76.9)	15 (55.6)	118 (80.8)	
- High grade	40 (23.1)	12 (44.4)	28 (19.2)	
**Tumour recurrence, *n* (%)**	56 (32.4)	8 (29.6)	48 (32.9)	0.740
**Cancer treatment, *n* (%)**				
- Radiotherapy	53 (30.6)	10 (37.0)	43 (29.5)	0.432
- Chemotherapy	20 (11.6)	5 (18.5)	15 (10.3)	0.218
- Temozolomide	16 (9.2)	4 (14.8)	12 (8.2)	0.277
**Surgery, *n* (%)**				
- Surgical biopsy	8 (4.6)	2 (7.4)	6 (4.1)	0.454
- Subtotal resection	34 (19.7)	9 (33.3)	25 (17.1)	0.052
- Gross total resection	131 (75.7)	16 (59.3)	115 (78.8)	0.030
**Treatment received, *n* (%)**				
- Steroids	139 (80.3)	20 (74.1)	119 (81.5)	0.372
- Antiepileptic drugs	100 (57.8)	22 (81.5)	78 (53.4)	0.007
**Acute hospital stay, mean (SD)**	21.5 (± 24.8)	42.8 (± 45.9)	17.6 (± 15.9)	0.009
**Inpatient rehabilitation length of stay, mean (SD)**	26.5 (± 22.4)	35.0 (± 42.8)	24.9 (± 15.8)	0.236
**Admission functional status, mean (SD)**				
FIM motor	47.5 (± 18.1)	33.5 (± 14.8)	50.1 (± 17.4)	< 0.001
- FIM self-care	24.3 (± 8.71)	17.2 (± 8.04)	25.6 (± 8.21)	< 0.001
- FIM sphincter	7.00 (± 4.42)	3.89 (± 3.87)	7.58 (± 4.29)	< 0.001
- FIM transfer	11.03 (± 4.39)	8.15 (± 3.77)	11.6 (± 4.29)	< 0.001
- FIM locomotion	5.24 (± 3.01)	4.22 (± 2.21)	5.43 (± 3.11)	0.019
FIM cognition	23.1 (± 9.24)	19.4 (± 9.99)	23.8 (± 8.96)	0.022
- FIM communication	10.2 (± 4.22)	8.41 (± 4.41)	10.5 (± 4.11)	0.016
- FIM social cognition	12.9 (± 5.57)	11.0 (± 5.92)	13.3 (± 5.45)	0.050
**Discharge functional status, mean (SD)**				
FIM motor	67.0 (± 19.0)	57.5 (± 19.6)	68.4 (± 18.6)	0.014
- FIM self-care	32.1 (± 8.26)	28.5 (± 8.84)	32.6 (± 8.08)	0.032
- FIM sphincter	10.5 (± 4.38)	9.29 (± 4.35)	10.6 (± 4.37)	0.203
- FIM transfer	15.6 (± 4.59)	13.2 (± 4.55)	15.9 (± 4.51)	0.012
- FIM locomotion	8.9 (± 3.62)	6.48 (± 3.52)	9.26 (± 3.50)	0.001
FIM cognition	27.4 (± 7.64)	23.7 (± 9.48)	27.9 (± 7.22)	0.065
- FIM communication	11.7 (± 3.51)	9.81 (± 3.87)	11.9 (± 3.38)	0.010
- FIM social cognition	15.7 (± 5.00)	13.9 (± 5.79)	16.0 (± 4.84)	0.077

ACUR: Acute Care Unit Readmissions; SD: Standard Deviation; ECOG: Eastern Cooperative Oncology Group; FIM: Functional Independence Measure.

In the study population, 133 (76.9%) patients had low-grade primary brain tumours (WHO I and II), and 40 (23.1%) patients had high-grade primary brain tumours (WHO III and IV). There were 32 patients diagnosed with glioblastoma multiforme, of which 25 (78.1%) patients were of the isocitrate dehydrogenase-wildtype, and 13 (40.6%) patients were of methylguanine-DNA methyltransferase methylated type. There were 56 (32.4%) patients who presented with tumour recurrence, of which 42 (24.3%) patients had low-grade primary brain tumours (WHO I and II), and 14 (8.1%) patients had high-grade primary brain tumours (WHO III and IV).

A majority of patients received steroids (80.3%) and antiepileptic drugs (57.8%) during their hospitalization stay. With regard to treatment, 53 (30.6%) received radiotherapy, 20 (11.6%) received chemotherapy and all patients underwent surgery either in the form of biopsy (4.6%), partial resection (19.7%) or near total resection (75.7%). There were 27 (15.9%) patients who had unplanned ACUR during the course of inpatient rehabilitation.

The causes of unplanned ACUR are shown in Table II. Neurosurgical complications (10) and infection (7) were the 2 most common reasons. None had > 1 unplanned ACUR.

**Table II:** Causes of unplanned Acute Care Unit Readmissions (*n* = 27)

Causes	*n* = 27
Neurosurgical complications, *n* (%)	10 (37.0)
Disease progression, *n* (%)	4 (14.8)
Non neurosurgical infections, *n* (%)	7 (25.9)
Seizures, *n* (%)	4 (14.8)
Cardiac, *n* (%)	2 (7.4)

Results of multivariate regression analysis are shown in Table III. This revealed that the significant factors for unplanned ACUR were a longer acute hospitalization stay (odds ratio = 1.024; 95% confidence interval [CI]1.01–1.04; *p* = 0.007), whereas a higher admission motor FIM was protective against unplanned ACUR (odds ratio = 0.945; 95% CI = 0.915–0.977; *p* = 0.001). Factors that were not significant were age, sex, ethnicity, marital status, ECOG performance status, lesion side, lesion location, lesion size, grade, tumour recurrence, radiotherapy, chemotherapy, surgical treatment, treatment received during hospitalization (steroids and antiepileptic drugs), rehabilitation length of stay and admission FIM cognition scores (Table III).

**Table III:** Regression analysis for associations with unplanned ACUR

Variable	Odds ratio (95% CI)	*p*
Age	0.962 (0.901–1.03)	0.239
Female sex	0.790 (0.215–2.90)	0.723
Ethnicity		
- Chinese	Reference	Reference
- Malay	0.210 (0.030–1.46)	0.114
- Indian	0.229 (0.007–7.83)	0.414
Marital status		
- Single	Reference	Reference
- Married	2.15 (0.454–19.25)	0.336
Initial ECOG		
-0	Reference	Reference
-1	1.07 (0.259–4.41)	0.927
Laterality		
- Left	Reference	Reference
- Right	0.383 (0.083–1.77)	0.219
- Bilateral	1.14 (0.173–7.58)	0.889
Lesion site		
- Cerebral	Reference	Reference
- Infratentorial	2.26 (0.411–12.36)	0.349
- Skull base	0.39 (0.640–1.24)	0.165
Lesion size (cm)		
- < 3	Reference	Reference
- 3–6	4.67 (0.728–30.0)	0.104
- > 6 cm	7.53 (0.776–73.0)	0.082
Grade		
- Low grade	Reference	Reference
- High grade	2.17 (0.342–13.74)	0.411
Tumour recurrence	1.00 (0.22–4.51)	0.999
Radiotherapy	0.590 (0.150–2.32)	0.450
Chemotherapy	0.683 (0.080–5.81)	0.727
Surgery		
- No surgery	Reference	Reference
- Surgical biopsy	0.065 (0.002–2.67)	0.149
- Partial resection	0.498 (0.023–10.7)	0.656
- Near total resection	0.037 (0.002–1.20)	0.124
Antiepileptic drugs	4.13 (0.88–19.2)	0.070
Steroids	2.31 (0.41–13.0)	0.341
Acute hospital stay length	1.024 (1.01–1.04)	0.007*
Inpatient rehabilitation length of stay	1.01 (0.987–1.03)	0.464
Admission FIM motor score	0.945 (0.915–0.977)	0.001*
Admission FIM cognitive score	1.07 (0.98–1.17)	0.138

ACUR: Acute Care Unit Readmissions; CI: Confidence Interval; ECOG: Eastern Cooperative Oncology Group; FIM: Functional Independence Measure. *p* < 0.05.

## DISCUSSION

Unplanned ACUR from rehabilitation facilities to acute care units signifies critical episodes necessitating acute medical attention, which demonstrates the vulnerabilities faced by primary brain tumour patients during their recovery phase.

We report an unplanned ACUR rate of nearly 15.9% of patients throughout their rehabilitation stay. The unplanned ACUR rate is similar to the 14.6% reported in a recent Korean study of patients with brain tumour undergoing intensive inpatient rehabilitation (6). In comparison, studies in patients with brain tumours in Western rehabilitation centres have also comparable reported incidence rates with unplanned ACUR of 17–35% or higher (5, 8, 9).

We found that the unplanned ACUR rate was significantly driven by neurosurgical and infective complications, although tumour progression and seizures are also common, indicating the complexity and challenges associated with managing brain tumours, especially in a rehabilitation setting. Neurosurgical complications may only occur at a several weeks after the acute episode of admission; hence, these findings suggest that surveillance and close monitoring of neurosurgical complications during inpatient rehabilitation are essential even after successful tumour resection and transfer to inpatient rehabilitation (10).

Examples of these neurosurgical conditions include postoperative haemorrhage or surgical site infections, which necessitate a high index of suspicion during rehabilitation as they require immediate medical attention. Patients undergoing rehabilitation are also similarly vulnerable to hospital-associated infections, such as pneumonia, urinary tract infection, venous thromboembolism and decubitus ulcers, which necessitates meticulous nursing care, the removal of unnecessary catheters and early mobilization (11). A study by Alam et al. found similar results, with infection being the primary cause of unplanned ACUR in cancer patients, which contrasts with non-cancer patients undergoing rehabilitation, for which cardiopulmonary factors account for most unplanned ACUR (5).

As patients may remain in rehabilitation for a few weeks to months, aggressive primary brain tumours may display clinical signs of progression such as motor weakness or worsening cognition. Hence, judicious selection of patients with aggressive brain tumours and candid discussions of rehabilitation goals may be necessary to avoid unnecessary unplanned ACUR in patients with tumour progression (9). Seizures are also common after brain tumour surgeries, and this should be monitored during inpatient rehabilitation (12).

We found that the length of acute admission and motor FIM were independent factors associated with unplanned ACUR occurrence. Our findings concur with Guo et al. who found that cancer patients who were transferred back to acute care had significantly longer acute hospital stays, with the authors hypothesizing that this may indicate a deterioration of health (9). This may indicate reduced physiological reserves in these patients, placing them at higher risk of developing complications requiring transfer back to acute care. However, due to the low ACUR occurrence, cautious interpretation of these findings is warranted, and further investigation in future studies is required.

Prolonged acute care stays may suggest underlying complications or a more complicated recovery trajectory for these patients, leading to higher probability of unplanned ACUR during rehabilitation. Studies have shown that an extended acute hospital length of stay likely indicates more acute postoperative complications, more vulnerable baseline patient characteristics, more comorbidities and poorer premorbid functional status (13).

A poorer motor function upon admission might signify a more unfavourable rehabilitation trajectory, increasing the likelihood of needing unplanned ACUR (14). For example, a lower motor FIM may indicate patients who are less mobile either from brain tumour-related impairments or treatment side effects, and more at risk of medical complications such as venous thromboembolism, atelectasis and urinary stasis. Supporting our findings is a study by Asher et al. in patients with cancer in an inpatient rehabilitation facility, which demonstrated that lower motor FIM alone was the best predictor of transferring patients with cancer from inpatient rehabilitation back to acute care (15). Our study found higher motor FIM score to be mildly protective towards a lower unplanned ACUR.

Several limitations of this study should be highlighted. First, the retrospective nature of the study meant that certain patient information was not available (e.g. the presence of medical complications occurring acute hospital stay and comorbidities), which may predispose patients to medical deterioration during rehabilitation. Second, it should be noted that this study reflects a single freestanding acute rehabilitation institution that is not co-located with the referring academic medical centre, and this may limit generalizability of the study findings to specific inpatient rehabilitative settings. Third, this study was also based on medical records collected over 7 years, and different criteria to select rehabilitation candidates may have changed over time. Fourth, although prolonged acute hospitalization was associated with unplanned ACUR in this study, we also did not capture the causes for acute hospitalization.

In conclusion, this study sheds light on the incidence, reasons and associations associated with unplanned ACUR amongst cancer patients undergoing acute inpatient rehabilitation. Patients with primary brain tumours undergoing rehabilitation have a high risk of unplanned ACUR for various reasons, including neurosurgical complications and infections. Patients with primary brain tumours should undergo careful rehabilitative assessment prior to rehabilitation admission, and patients and their family should be counselled accordingly about the complications and unplanned ACUR risks. Additionally, these patients require vigilant monitoring throughout their rehabilitation journey, early detection of medical complications and adequate education of involved rehabilitation professionals in the recognition and management of these complications. Further prospective studies with larger cohorts are warranted to validate these findings and develop targeted interventions aimed at reducing unplanned ACUR and optimizing rehabilitation outcomes for primary brain tumour patients.
